# Psychostimulant Effect of the Synthetic Cannabinoid JWH-018 and AKB48: Behavioral, Neurochemical, and Dopamine Transporter Scan Imaging Studies in Mice

**DOI:** 10.3389/fpsyt.2017.00130

**Published:** 2017-08-04

**Authors:** Andrea Ossato, Licia Uccelli, Sabrine Bilel, Isabella Canazza, Giovanni Di Domenico, Micol Pasquali, Gaia Pupillo, Maria Antonietta De Luca, Alessandra Boschi, Fabrizio Vincenzi, Claudia Rimondo, Sarah Beggiato, Luca Ferraro, Katia Varani, Pier Andrea Borea, Giovanni Serpelloni, Fabio De-Giorgio, Matteo Marti

**Affiliations:** ^1^Department of Life Sciences and Biotechnology (SVeB), University of Ferrara, Ferrara, Italy; ^2^Section of Legal Medicine, Institute of Public Health, Catholic University of the Sacred Heart, Rome, Italy; ^3^Morphology, Surgery and Experimental Medicine Department, University of Ferrara, Ferrara, Italy; ^4^Physics and Hearth Science Department, University of Ferrara, Ferrara, Italy; ^5^Legnaro National Laboratories, Italian National Institute for Nuclear Physics (LNL-INFN), Legnaro, Italy; ^6^Department of Biomedical Sciences, University of Cagliari, Cagliari, Italy; ^7^Department of Medical Sciences, University of Ferrara, Ferrara, Italy; ^8^Department of Diagnostic and Public Health, University of Verona, Verona, Italy; ^9^Department of Psychiatry in the College of Medicine, Drug Policy Institute, University of Florida, Gainesville, FL, United States; ^10^Center for Neuroscience, Istituto Nazionale di Neuroscienze, Ferrara, Italy

**Keywords:** AKB48, cocaine, dopamine transporter, microdialysis, SPECT-CT imaging, JWH-018, synthetic cannabinoids, psychostimulants

## Abstract

JWH-018 and AKB48 are two synthetic cannabinoids (SCBs) belonging to different structural classes and illegally marketed as incense, herbal preparations, or chemical supply for theirs psychoactive cannabis-like effects. Clinical reports from emergency room reported psychomotor agitation as one of the most frequent effects in people assuming SCBs. This study aimed to investigate the psychostimulant properties of JWH-018 and AKB48 in male CD-1 mice and to compare their behavioral and biochemical effects with those caused by cocaine and amphetamine. *In vivo* studies showed that JWH-018 and AKB48, as cocaine and amphetamine, facilitated spontaneous locomotion in mice. These effects were prevented by CB_1_ receptor blockade and dopamine (DA) D_1/5_ and D_2/3_ receptors inhibition. SPECT-CT studies on dopamine transporter (DAT) revealed that, as cocaine and amphetamine, JWH-018 and AKB48 decreased the [^123^I]-FP-CIT binding in the mouse striatum. Conversely, *in vitro* competition binding studies revealed that, unlike cocaine and amphetamine, JWH-018 and AKB48 did not bind to mouse or human DAT. Moreover, microdialysis studies showed that the systemic administration of JWH-018, AKB48, cocaine, and amphetamine stimulated DA release in the nucleus accumbens (NAc) shell of freely moving mice. Finally, unlike amphetamine and cocaine, JWH-018 and AKB48 did not induce any changes on spontaneous [^3^H]-DA efflux from murine striatal synaptosomes. The present results suggest that SCBs facilitate striatal DA release possibly with different mechanisms than cocaine and amphetamine. Furthermore, they demonstrate, for the first time, that JWH-018 and AKB48 induce a psychostimulant effect in mice possibly by increasing NAc DA release. These data, according to clinical reports, outline the potential psychostimulant action of SCBs highlighting their possible danger to human health.

## Introduction

According to the European Drug Report, 100 new abused substances have been detected for the first time in 2016 (1). Recent literature reported that an incredibly huge number of synthetic cannabinoids (SCBs) has been detected and commonly abused in the US, Europe, and Australia as Marijuana substitutes (2). Indeed, they are not preferred over cannabis but recreationally used to circumvent legal, work- and cost-related obstacles.

The consumption of SCBs can cause adverse events that directly jeopardize the subjects’ lives or promote harmful consequences as agitation, tachycardia, sudden cardiac arrest, and seizures along with liver and kidney failure. Suicide and self-injury have also been reported in individuals consuming SCBs (3).

JWH-018 (1-pentyl-3-(1-naphthoyl)indole) and AKB48 (*N*-(1-adamantyl)-1-pentyl-1H-indazole-3-carboxamide), respectively, classified as naphthoylindoles and adamantylindazoles, have been seized in different countries (4, 5). *In vitro* binding studies shown that JWH-018 and AKB48 display nanomolar affinity for both CD-1 murine and human CB_1_ and CB_2_ receptors, presenting a slight preference for CB_2_ receptors (6, 7). In particular, in CD-1 murine preparation, AKB48 and JWH-018 displayed a similar affinity for CB_1_ receptors [Ki = 5.34 and 5.82 nM, respectively; (6)], while AKB48 showed a slightly higher affinity than JWH-018 [Ki = 9.53 and 3.24 nM, respectively; (6)] for human CB_1_ receptors. Based on these findings, it seems likely that, compared to other SCBs, the two compounds might induce similar or higher *in vivo* effects.

CB_1_ receptors are highly expressed as limbic regions, such as the ventral tegmental area (VTA), the nucleus accumbens (NAc), ventral pallidum and prefrontal cortex (PFC). SCBs probably act in these brain regions by modulating reward, addiction, and cognitive functions (8). In line with this view, several rodent studies showed that these compounds, similar to other drugs of abuse, affect the mesolimbic dopaminergic transmission (7, 9, 10) and influence conditioned behaviors (11, 12).

It has been reported that SCBs may have atypical side effects, often larger and more negative than those of natural cannabinoids. For example, as detected by National Poison Data System that tracks US poison control calls, agitation is the most common adverse effect of SCBs consumption observed in humans (3), while other reported side effects are irritability, sadness, restlessness, aggression, combativeness, and psychomotor agitation (13–15). Differently, high doses of Δ^9^-THC or cannabis intoxication can cause, among other adverse events, xerostomia, injected conjunctivae, tachycardia, and psychotic effects (including hallucinations and paranoia) (14). Extreme agitation, irritability physical violence, convulsions, and nephrotoxicity have also been reported after SCBs consumption (16). Preclinical data have reported that JWH-018 (17), AKB48 (7) and other SCBs (7) increase, in a narrow range of doses, spontaneous locomotion in mice. This behavioral effect resembles the psychostimulant action of cocaine (18–22) and amphetamine (23–25). Moreover, previous *in vivo* microdialysis studies demonstrated that JWH-018, at the dose of 0.25 mg/kg i.p. [but not at lower (0.125 mg/kg i.p.) or higher (0.5 mg/kg i.p.) doses], increases dopamine (DA) transmission in the NAc shell but not in the NAc core and in the mPFC (9). Similar pharmacological properties were displayed by subsequent chemical generations of SCBs (7, 10, 26). However, the mechanism of action of JWH-018, AKB48, and their analogs is still not completely understood.

This study, by combining different experimental approaches, such as *in vitro* (binding), *in vivo* (behavioral tests, imaging and microdialysis) and *ex vivo* (synaptosome) ones, aimed at clarifying how these SCBs modulate dopaminergic signaling and whether these putative effects could be relevant for their locomotion facilitating properties. In particular, the effects of JWH-018 and AKB48 have been compared to those induced by cocaine and amphetamine, two psychostimulant drugs affecting the dopamine transporter (DAT) in a different way. Indeed, while cocaine acts as a DAT blocker by directly binding to DAT and, thus, preventing the translocation of DA, amphetamine competes with DA for binding to the empty transporter, leading to the reverse transport (efflux) of DA from the intracellular compartment to the synaptic cleft, thus exerting indirect effects [e.g., it reverses the action of VMAT2; (27)]. In view of the results obtained, the involvement of CB_1_ receptor- and the D_1_/D_2_ receptor-mediated mechanisms in the behavioral effects induced by JWH-018 and AKB48 has also been evaluated.

## Materials and Methods

### Animals

Male ICR (CD-1^®^) mice, 25–30 g (Harlan Italy; S. Pietro al Natisone, Italy), were group-housed (8–10 mice per cage; floor area per animal was 80 cm^2^; minimum enclosure height was 12 cm) on a reverse12:12-h light-dark cycle, temperature of 20–22°C, and humidity of 45–55%; and were provided *ad libitum* access to food (Diet 4RF25 GLP; Mucedola, Settimo Milanese, Milan, Italy) and water. The experimental protocols performed in this study were in accordance with the new European Communities Council Directive of September 2010 (2010/63/EU) a revision of the Directive 86/609/EEC and were approved by the Italian Ministry of Health and by the Ethical Committee of the University of Ferrara and of the University of Cagliari (*microdialysis studies*). Moreover, adequate measures were taken to minimize the number of animals used and their pain and discomfort.

### Drug Preparation and Dose Selection

Amphetamine sulfate, cocaine, ketamine hydrochloride, JWH-018, and AKB48 were purchased from LGC Standards (LGC Standards S.r.L., Sesto San Giovanni, Milan, Italy), xylazine hydrochloride from Sigma-Aldrich (St. Louis, MO, USA) and GBR 12783 dihydrochloride, AM-251, SCH23390, and haloperidol from Tocris (Bristol, United Kingdom).

For *in vivo* behavioral studies, all compounds (JWH-018, AKB48, amphetamine sulfate, cocaine hydrochloride, AM-251, SCH23390, and haloperidol) were initially dissolved in absolute ethanol and Tween 80 and then diluted to the final volume with saline (0.9% NaCl; final ethanol or Tween 80 concentration = 2%) The ethanol, Tween 80, and saline solution were also used as vehicle. Drugs were administered by intraperitoneal injection in a volume of 4 μl/g. The used doses of JWH-018 (0.3 and 1 mg/kg i.p.) and AKB48 (0.3 and 1 mg/kg i.p.) were chosen based on previous studies (6, 7, 9, 10).

For *in vitro* release experiments, JWH-018 and AKB48 were dissolved in absolute ethanol (ethanol = vehicle; maximum concentration = 0.04% v/v). The used concentrations of JWH-018, AKB48, cocaine, and amphetamine were chosen on the basis of previous studies (7, 17, 28, 29). Moreover, for *in vivo* DaTSCAN, imaging studies, the [^123^I]-FP-CIT (^123^I-2β-carbomethoxy-3β-(4-iodophenyl)-*N*-(3-fluoropropyl)nortropane, [^123^I]-IDaTSCAN) was purchased from GE Healthcare B.V. Den Dolech 2 NL-5612 AZ, Eindhoven, The Netherlands (specific activity 2.5–4.5 × 10^14^ Bq/mmol at the date and time of calibration; radiochemical purity >97%).

### Spontaneous Locomotor Activity

The spontaneous locomotor activity was measured by using the ANY-maze video tracking system (Ugo Basile, application version 4.99 g Beta). The mouse was placed in a square plastic cage (60 cm × 60 cm) located in a sound- and light-attenuated room and motor activity was monitored for 240 min. Four mice were monitored in parallel in each experiment. Parameters measured were distance traveled (meter), total time in the peripheral zone (seconds), total time in the central zone (seconds), and immobility time (seconds; the animal was considered immobile when 95% of his image remained in the same place for at least 2 s). Parameters were analyzed every 15 min for a maximum of 240 min and to avoid mice olfactory cues, cages were carefully cleaned with a dilute (5%) ethanol solution and washed with water between animal trials. All experiments were performed between 9:00 a.m. and 1:00 p.m.

### *In Vivo* DaTSCAN, Imaging Studies

SPECT-CT studies have been performed using a YAP(S)PET scanner (30–33). The spatial resolution of the system was verified for ^123^I, using a NEMA NU 4-2008 phantom (34) with hot rods ranging from 1 to 5 mm. 18 CD-1 male mice were divided into six different groups (three mice per treatment). During the scanning procedure, each mouse was previously anesthetized by intramuscular injections of a mixture of ketamine and xilazine (respectively, 100 and 20 mg/kg), and submitted to a pretreatment (by intraperitoneal injection) with vehicle (see drug preparation and animal dose determination), cocaine (20 mg/kg), amphetamine sulfate (10 mg/kg), JWH-018 (1 mg/kg), or AKB48 (1 mg/kg). A control group (i.e., naïve untreated mice) was also included in the study. Thirty minutes after drug administration, all mice were submitted to an intravenous injection with a solution of [^123^I]-DaTSCAN (15–20 MBq, ≤200 µl). The body temperature of the animals was maintained at 37°C during the imaging sessions and under the cage, between imaging sessions, using a heating lamp. The SPECT–CT whole-body images were acquired at 1 h and 30 min after [^123^I]-FP-CIT injection, with the initial tomographic acquisition starting nearly 15 min after the injection. Each SPECT-CT whole-body acquisition consisted of one bed positions (36 mm), 60 min, 128 views over 360 (35). The used energy window is 119–219 keV and the images were reconstructed by using the iterative EM-ML algorithm, including the collimator response. CT images have been acquired, using the digital X-ray imaging system integrated into the YAP(S)PET scanner (36). Acquisition parameters for X-ray projections were X-ray tube voltage = 35 keV, anode current = 1 mA, exposure = 1 s, 64 views over 360, and magnification factor = 1.2. Subtraction of dark noise contribution and flat field corrections was accomplished to obtain final images. The CT data were reconstructed by using the FDK algorithm. Amide software (37) has been used for images’ registration, visualization and analysis. The size of the ROIs was voxels (100 mm^3^ volume), corresponding to entire striatum. These ROIs were used as a template. To avoid the variability of the slice selection and to gain statistical power, the entire striatum volume for the analysis was used. The template was positioned manually (without changing the size and form of the ROIs) on the SPECT images with the backing of anatomical information from LONI MAP 2003 MRI mouse atlas (38, 39). For analysis of striatal [^123^I]-FP-CIT binding, two consecutive horizontal slices (total thickness approximately 4 mm) with the highest striatal binding were selected. The landmarks for positioning were the intra-orbital glands, striatum, and the borders of the brain. Striatal binding ratios are expressed as average activity per unit volume [Bq/mm^3^], each value has been calculated as the ratio between the activity inside the ROI and the ROI volume, normalized for injected activity and for mouse brain weight.

### [^3^H]-WIN 35,428 Competition Binding Experiments

Competition binding experiments were carried out incubating 8 nM [^3^H]-WIN 35,428 (specific activity 84 Ci/mmol; Perkin Elmer, Boston, MA, USA) with CHO membranes transfected with human DAT (Perkin Elmer) or mouse striatal synaptosomes with different concentration of the examined compounds for 120 min at 4°C. Non-specific binding was determined in the presence of 1 µM GBR 12783. At the end of the incubation time, bound and free radioactivity were separated by filtering the assay mixture through Whatman GF/B glass fiber filters in a Brandel cell harvester (Brandel, Unterföhring, Germany). Filter bound radioactivity was counted in a Perkin Elmer 2810TR scintillation counter (Perkin Elmer).

### *In Vivo* Brain Microdialysis Studies

Male ICR (CD-1^®^) mice, 25–30 g (ENVIGO. Harlan Italy; S. Pietro al Natisone, Italy) were anesthetized with Isoflurane (3%; 200 ml/min) and implanted with vertical dialysis probe (1 mm dialyzing portion) prepared with AN69 fibers (Hospal Dasco, Bologna, Italy) in the NAc shell (A + 1.4, L 0.4 from bregma, V-4.8 from dura) according to the mouse brain atlas by Paxinos and Franklin (40). On the day following surgery, probes were perfused with Ringer’s solution (147 mM NaCl, 4 mM KCl, 2.2 mM CaCl_2_) at a constant rate of 1 µl/min. Dialyzate samples (10 µl) were injected into an HPLC equipped with a reverse phase column (C8 3.5 um, Waters, USA) and a coulometric detector (ESA, Coulochem II) to quantify DA. The first electrode of the detector was set at +130 mV (oxidation) and the second at −175 mV (reduction). The composition of the mobile phase was as follows: 50 mM NaH_2_PO_4_, 0.1 mM Na_2_-EDTA, 0.5 mM n-octyl sodium sulfate, 15% (v/v) methanol, pH 5.5. The sensitivity of the assay for DA was 5 fmol/sample. At the end of each experiment, animals were sacrificed and their brains removed and stored in formalin (8%) for histological examination to verify the correct placement of the microdialysis probe.

### Striatal Synaptosome Preparation

On the day of the experiment, the animal was euthanized, the brain was rapidly removed, and both striata isolated. Thereafter, a crude synaptosomal (P2) fraction was prepared as follows: the striata were suspended in ice-cold buffered sucrose solution (0.32 M, pH 7.4) and homogenized. The homogenate was centrifuged (10 min, 2,100 *g*, 4°C) to remove nuclei and debris. The supernatant was further centrifuged at 13,500 *g* for 20 min at 4°C. For [^3^H]-WIN 35,428 binding experiments, the P2 pellet was resuspended in 50 mM Tris–HCl, 100 mM NaCl, pH 7.4. For [^3^H]-DA release experiments, the P2 pellet was then resuspended in 5 ml of Kreb’s solution (mM: NaCl 118; KCl 4.4; CaCl_2_ 1.2; MgSO_4_ 1.2; KH_2_PO_4_ 1.2; NaHCO_3_ 25; glucose 10), gassed 20 min with a mixture of 95% O_2_ plus 5% CO_2_ containing [^3^H]-DA (50 nM; Perkin Elmer, Monza, Italy), disodium EDTA (0.03 mM), and ascorbic acid (0.05 mM; to prevent [^3^H]-DA degradation).

### Spontaneous [^3^H]-DA Release

After synaptosomal preparation, 0.5 ml aliquots of the suspension were distributed on microporous filters placed at the bottom of a set of parallel superfusion chambers maintained at 37°C and perfused with aerated (95% O_2_/5% CO_2_) Kreb’s solution (0.3 ml/min). After 30 min of superfusion to equilibrate the system, 5-min fractions were collected from the 30th to the 75th min (nine samples). When required, after the collection of three basal samples, amphetamine (10 µM), cocaine (100 nM), JWH-018 (100 nM, 1 µM), AKB48 (100 nM, 1 µM), and vehicle were added to the perfusion solution in order to evaluate their effects on spontaneous [^3^H]-DA release. At the end of the experiment, the radioactivity of the samples and filters was determined by liquid scintillation spectrometry (LS1800 Beckman). In view of the results obtained, in a separate set of experiments, [^3^H]-DA uptake was also evaluated.

### [^3^H]-DA Uptake Experiments

After synaptosomal preparation, the suspension was maintained under a light and continuous oxygenation (95% O_2_, 5% CO_2_) for 20 min at 37°C. Thereafter, 0.5 ml aliquots of striatal synaptosomal suspension were prepared. When required the selective DA reuptake blocker GBR 12783 (100 nM, Sigma-Aldrich, USA), cocaine (100 nM), amphetamine (1 µM), JWH-018 and AKB048 (100 nM, 1 µM), and vehicle were added and after 5 min the synaptosomes were incubated for 10 min with 50 nM [^3^H]-DA. After this period, the reaction was stopped by filtration through microporus nylon filters (0.45 µm, 13 mm; Analytical Technology, Brugherio, Italy). The filters were then washed with 1 ml ice-cold Kreb’s solution and the radioactivity accumulated on synaptosomes was extracted by eluting two times with 1 ml of warm NaOH (1 N) and then determined by liquid scintillation spectrometer. Non-specific uptake was measured by following the same procedure at 0°C.

## Results

### Studies on Spontaneous Locomotor Activity in Mice

**Figure 1:**
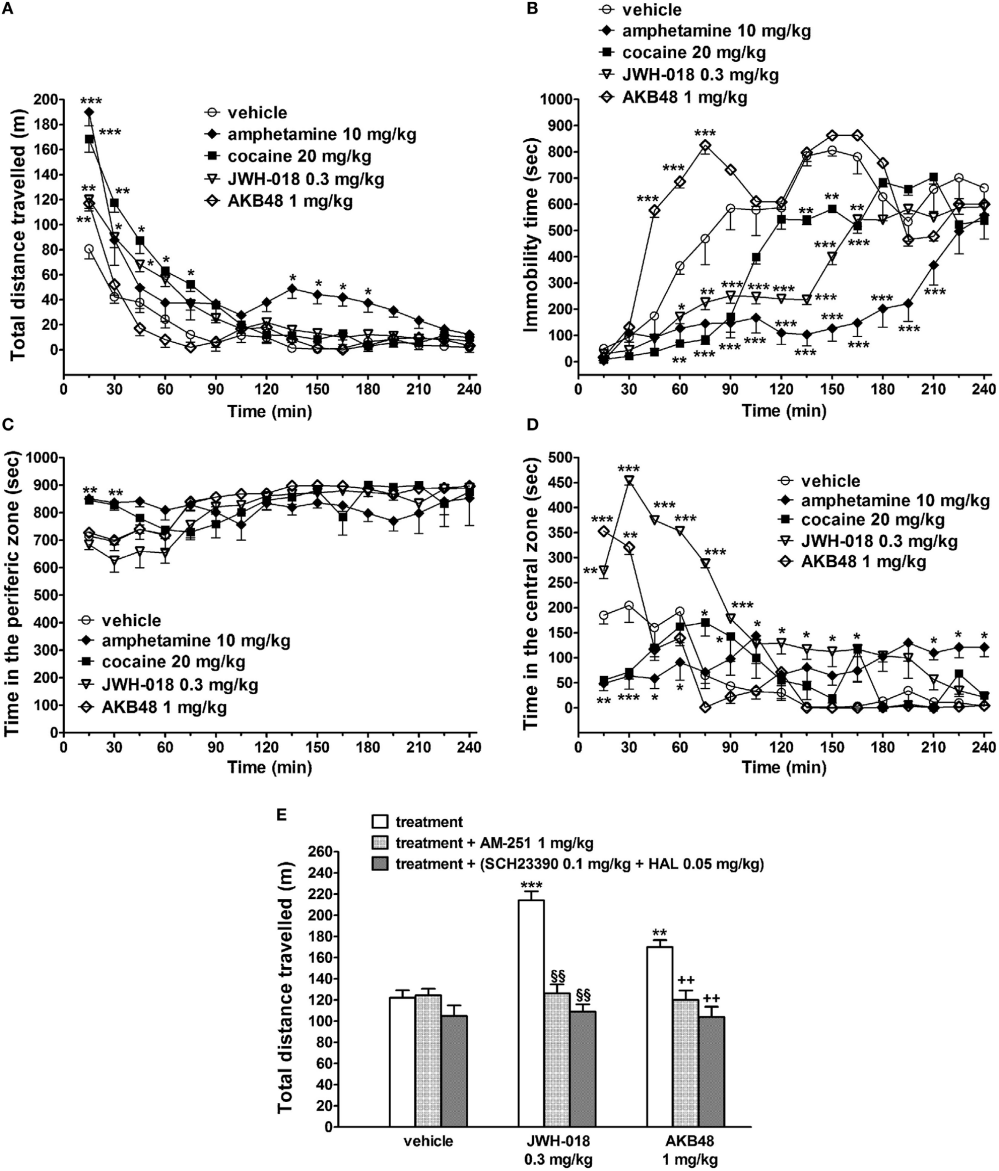
Effect of the systemic administration of vehicle, amphetamine (10 mg/kg i.p.), cocaine (20 mg/kg i.p.), JWH-018 (0.3 mg/kg i.p.), and AKB48 (1 mg/kg i.p.) on the total distance traveled **(A)**, on the immobility time **(B)**, and on the total time spent in the peripheral and central area **(C,D)** of the mouse. Interaction of JWH-018 and AKB48 with the selective CB_1_ receptor antagonist AM 251 [6 mg/kg, i.p.; **(E)**], the D_1_ receptor antagonist SCH23390 [0.1 mg/kg i.p.; **(E)**], and the D_2_ receptor antagonist haloperidol [HAL; 0.05 mg/kg i.p.; **(E)**]. AM 251, and SCH23390 + HAL were administered 20 min before synthetic cannabinoids injection. Data are expressed as meters (total distance traveled) and as seconds (immobility time; time in the peripheral and central zone). Data represent the mean ± SEM of eight determinations for each treatment. Statistical analysis was performed by two-way ANOVA followed by Bonferroni’s test for multiple comparisons **(A–D)** or by one-way ANOVA followed by Tukey’s test **(E)**. *p < 0.05, **p < 0.01, ***p < 0.001 versus vehicle; ^§§^*p* < 0.01 versus JWH-018; ^++^*p* < 0.01 versus AKB48.

The acute i.p. administration of JWH-018 (0.3 mg/kg), amphetamine (10 mg/kg), and cocaine (20 mg/kg) induced long-lasting increases in the total distance traveled (i.e., spontaneous locomotion) by the mice, while AKB48 (1 mg/kg) facilitated the spontaneous locomotion only in the first 15 min after the injection [Figure 1A; significant effect of treatment (*F*_4,560_ = 64.65, *p* < 0.0001), time (*F*_15,560_ = 120.40, *p* < 0.0001), and time × treatment interaction (*F*_60,560_ = 4.628, *p* < 0.0001)]. In particular, the effects of JWH-018 or cocaine lasted 90 min, while amphetamine increased the mouse spontaneous locomotion also from 135 to 210 min after drug administration.

JWH-018, amphetamine, and cocaine reduced the immobility time in mice, while AKB48 increased it 30 min after the drug administration [Figure 1B; significant effect of treatment (*F*_4,560_ = 199.3, *p* < 0.0001), time (*F*_15,560_ = 79.13, *p* < 0.0001), and time × treatment interaction (*F*_60,560_ = 10.39, *p* < 0.0001)]. Differently to mice treated with cocaine and amphetamine, JWH-018- and AKB48-injected animals spent more time in the central zone [Figure 1D; significant effect of treatment (*F*_4,560_ = 70.37, *p* < 0.0001), time (*F*_15,560_ = 32.48, *p* < 0.0001), and time × treatment interaction (*F*_60,560_ = 12.24, *p* < 0.0001)] than in the peripheral area of the cage [Figure 1C; significant effect of treatment (*F*_4,560_ = 9.751, *p* < 0.0001), time (*F*_15,560_ = 13.33, *p* < 0.0001), and time × treatment interaction (*F*_60,560_ = 4.394, *p* < 0.0001)].

The facilitation of spontaneous locomotion induced by JWH-018 (0.3 mg/kg) and AKB48 (1 mg/kg) was prevented by a pretreatment with AM 251 [1 mg/kg i.p.; Figure 1E: significant effect of treatment (*F*_3,56_ = 13.74, *p* < 0.0001), time (*F*_1,56_ = 31.88, *p* < 0.0001), and time × treatment interaction (*F*_3,56_ = 17.59, *p* < 0.0001)] or by the coadministration of SCH23390 (0.1 mg/kg i.p.) and haloperidol [0.05 mg/kg i.p.; Figure 1E: significant effect of treatment (*F*_3,56_ = 13.74, *p* < 0.0001), time (*F*_1,56_ = 31.88, *p* < 0.0001), and time × treatment interaction (*F*_3,56_ = 17.59, *p* < 0.0001)]. AM 251, SCH23390, and haloperidol by themselves did not alter the spontaneous locomotion in mice (Figure 1E).

### *In Vivo* DaTSCAN, Imaging Studies

**Figure 2:**
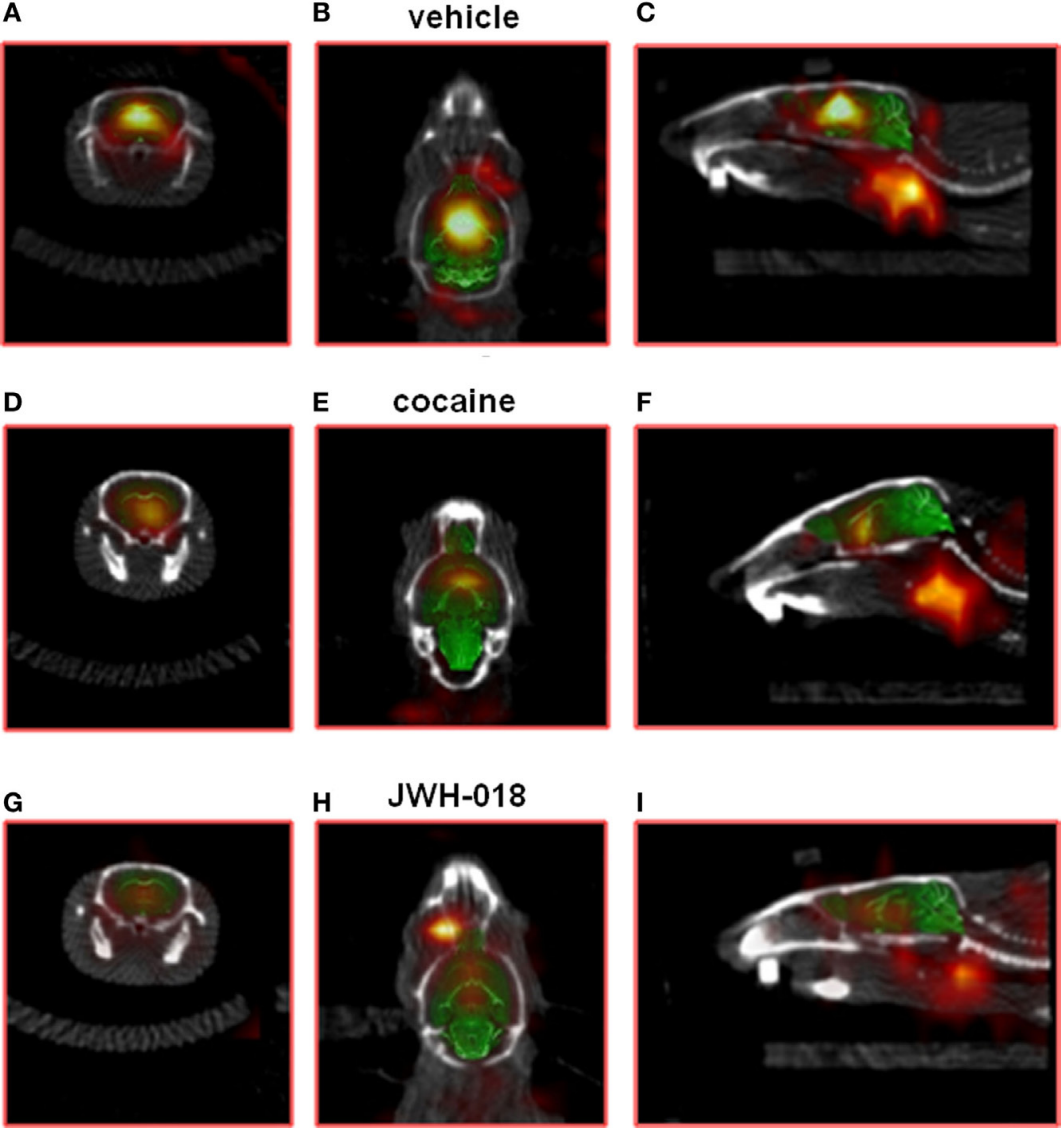
Sample slice from a [^123^I]-FP-CIT SPECT/CT image of a vehicle [**(A–C)**; respectively, coronal, transverse, and sagittal plan], cocaine [**(D–F)**; respectively, coronal, transverse, and sagittal plan], and JWH-018 [**(G–I)**; respectively, coronal, transverse, and sagittal plan] treated mice. ROIs for the striatum.

**Figure 3:**
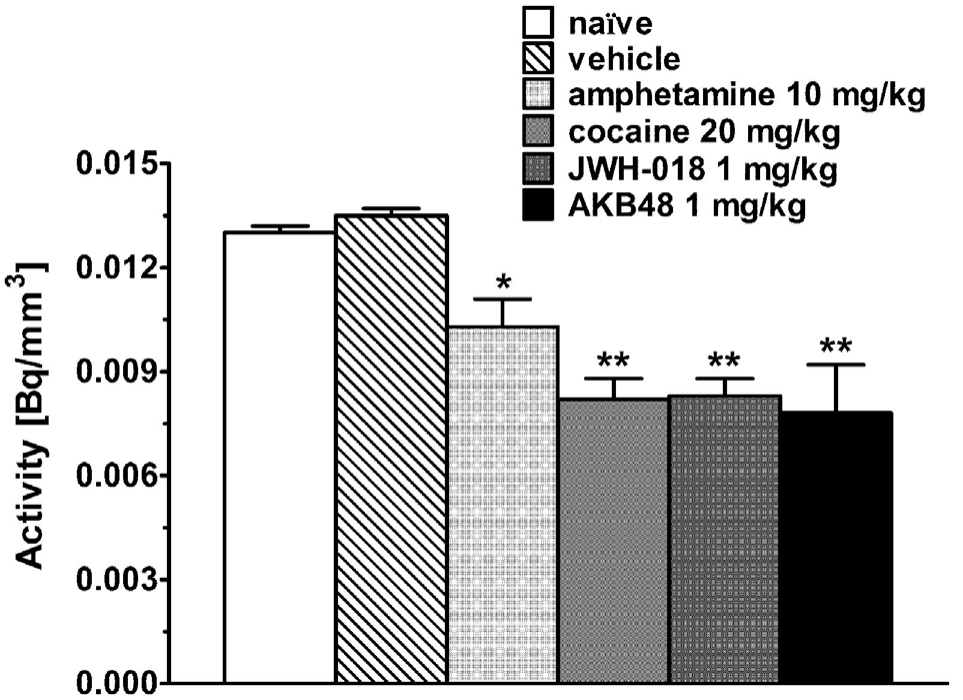
Striatal uptake of [^123^I]-FP-CIT in control mice (naïve) and in mice after the administration of vehicle, amphetamine (10 mg/kg), cocaine (20 mg/kg), JWH-018 (1 mg/kg), and AKB48 (1 mg/kg). Means and SDs are shown. Significance levels are *p < 0.05, **p < 0.01.

Intense, symmetrical [^123^I]-FP-CIT binding was observed in the striatum of control mice (*images not shown*). Vehicle injection did not change [^123^I]-FP-CIT binding in the striatum of mice (Figures 2A–C). The acute systemic injection of cocaine (20 mg/kg i.p.; Figures 2D–F) or amphetamine (10 mg/kg i.p.; *images not shown*) induced significant decreases of the [^123^I]-CIT binding in the striatum of mice (reduction of ~40 and ~25%, respectively; Figure 3). Similarly, the administration of JWH-018 (1 mg/kg i.p.; Figures 2G–I) or AKB48 (1 mg/kg i.p.; *images not shown*) decreased the [^123^I]-FP-CIT binding in the striatum of mice (reduction of ~39 and ~42%, respectively; Figure 3); these effects were comparable to those caused by the administration of cocaine (Figure 3).

### Competition Binding Experiments on Mice and Human DAT

Competition binding experiments with the reference compound GBR 12783 revealed that it displays a similar affinity for human and mouse DAT (Table 1). As expected, cocaine showed affinity for DAT in the nanomolar range, with Ki values of 174 and 193 nM in CHO membranes transfected with human DAT or mouse striatal synaptosomes, respectively. Amphetamine bound human and mouse DAT with affinity values of 554 and 622 nM, respectively. Interestingly, the SCBs JWH-018 and AKB48 were able to bind human DAT with affinity values of 7,183 and 4,588 nM, respectively (Table 1).

**Table 1:** Affinity values of GBR 12783, cocaine, amphetamine, JWH-018, and AKB48 to DAT obtained from [^3^H]-WIN 35,428 competition binding experiments in human CHO membranes transfected with DAT and in mouse striatal synaptosomes.

Compounds	hDAT-CHO membranes Ki (nM)	Mouse striatal synaptosomes Ki (nM)
GBR 12783	1.93 ± 0.14	1.72 ± 0.11
Cocaine	174 ± 13	193 ± 16
Amphetamine	554 ± 47	622 ± 53
JWH-018	7,183 ± 528	>10,000
AKB48	4,588 ± 326	>10,000

*Data are expressed as mean ± SEM*.

### *In Vivo* Microdialysis Study

**Figure 4:**
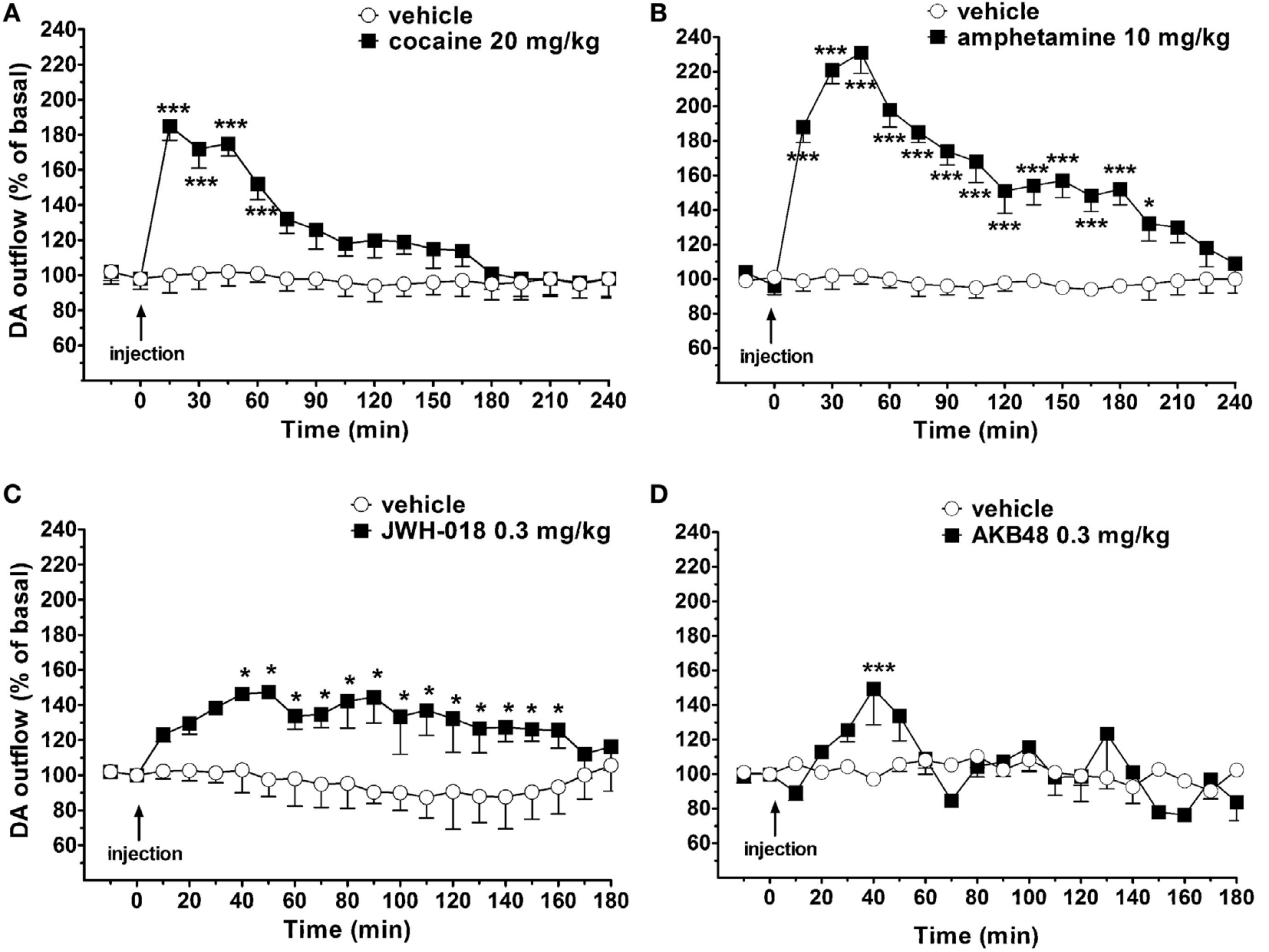
Effect of the systemic administration of cocaine [20 mg/kg i.p.; **(A)**], amphetamine [10 mg/kg i.p.; **(B)**], JWH-018 [0.3 mg/kg i.p.; **(C)**], and AKB48 [0.3 mg/kg i.p.; **(D)**] on dopamine (DA) transmission in the nucleus accumbens (NAc) shell of mice. Results are expressed as mean ± SEM of change in DA extracellular levels expressed as the percentage of basal values. *p < 0.05, ***p < 0.001 versus vehicle (NAc shell *n* = 13) (two-way ANOVA, Tukey’s HSD *post hoc*).

Basal NAc shell extracellular DA levels were 15 ± 5 fmol/10 μl sample. Systemic administration of amphetamine (10 mg/kg i.p.), cocaine (20 mg/kg i.p.), JWH-018 (0.3 mg/kg i.p.), and AKB48 (0.3 mg/kg i.p.) significantly increased NAc shell extracellular DA levels in the awake and freely moving mice (Figures 4A–D). Interestingly, JWH-018 or AKB48 had a different profile of action. In fact, JWH-018 induced a long-lasting increase of NAc shell extracellular DA levels (~150% of baseline values; Figure 4C), while AKB48 caused a rapid and significant increase in extracellular DA levels in the NAc shell of mice, reaching a peak value (~150% of baseline values) 40 min (Figure 4D) after its administration.

### Effects of Cocaine, Amphetamine, JWH-018, and AKB48 on Spontaneous [^3^H]-DA Release in Striatal Synaptosomes

**Figure 5:**
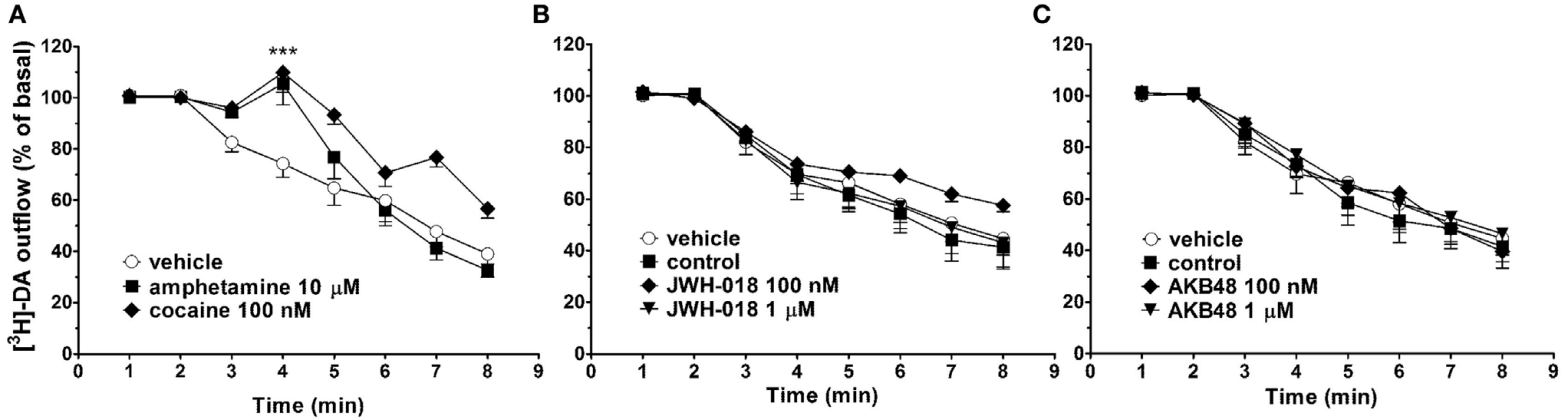
Effect of cocaine [100 nM; **(A)**], amphetamine [10 µM; **(A)**], JWH-018 [100 nM and 1 µM, **(B)**], and AKB48 [100 nM and 1 µM, **(C)**] on spontaneous [^3^H]-dopamine (DA) efflux from striatal synaptosomes obtained from CD-1 mice. Data are expressed as percentage of basal values and represent the mean ± SEM of 4–6 repetitions for each treatment. ***p < 0.001 significantly different from the respective control group according to ANOVA followed by Newman–Keuls test for multiple comparisons.

In synaptosomes from mouse striatum, spontaneous [^3^H]-DA efflux tended to decrease during the collection period (from 30 to 75 min from the start of perfusion, Figure 5). As expected, the perfusion with amphetamine (10 µM), or cocaine (100 nM), induced a significant increase in spontaneous [^3^H]-DA efflux from mouse striatal synaptosomes (Figures 5A–C). On the other hand, JWH-018 and AKB48 (100 nM and 1 µM) did not affect spontaneous [^3^H]-DA efflux from striatal synaptosomes (Figures 5B,C, respectively).

### Effects of Cocaine, Amphetamine, JWH-018, and AKB48 on [^3^H]-DA Uptake

**Figure 6:**
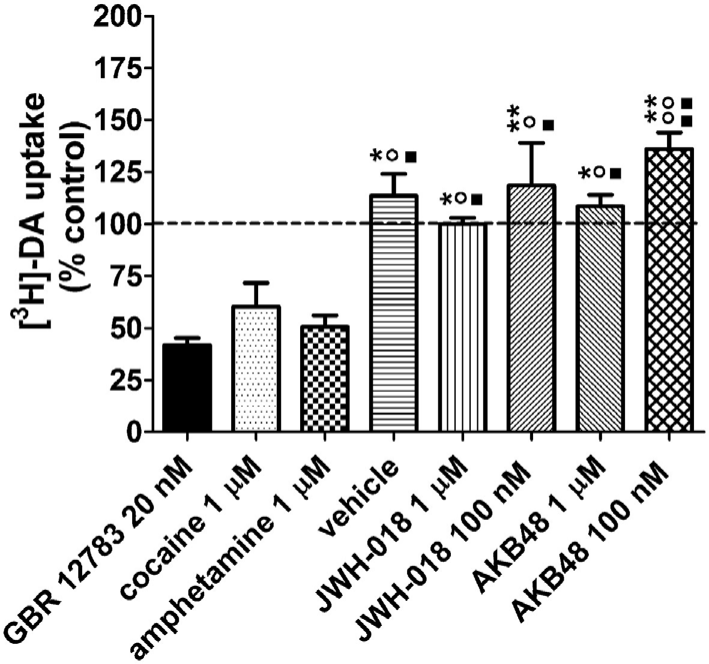
Effects of cocaine (1 µM), the selective dopamine (DA) reuptake blocker GBR 12783 (20 nM), amphetamine (1 µM), JWH-018 (1 µM, 100 nM), and AKB48 (1 µM, 100 nM) on [^3^H]-DA uptake in striatal synaptosomes from CD-1 mice. The drugs were added to synaptosomes 5 min before [^3^H]-DA and uptake was measured for 10 min at 37°C. A same volume of drug vehicle (Kreb’s solution or ethanol) was added 5 min before [^3^H]-DA incubation in the control/vehicle groups, respectively. The effect of the treatments on [^3^H]-DA uptake is expressed as percent of control values, i.e., tritium content measured in untreated synaptosomal aliquots, always assayed in parallel (100 ± 3%, *n* = 4; indicated by a dashed line). Unspecific uptake was measured at 0°C. Each treatment bar represents the mean ± SEM of four determinations ran in duplicate. **p < 0.01, *p < 0.05 significantly different from GBR 12783 20 nM; °°*p* < 0.01, °*p* < 0.05 significantly different from amphetamine 1 µM; ■■*p* < 0.01, ■*p* < 0.05 significantly different from cocaine 100 nM according to one-way ANOVA followed by Newman–Keuls test for multiple comparisons.

As shown in Figure 6, cocaine (1 µM) and amphetamine (1 µM) reduced [^3^H]-DA uptake in mouse striatal synaptosomes in the order of 50 and 40%, respectively. At 20 nM, GBR 12783 produced a similar inhibition of [^3^H]-DA uptake as found with 100 nM of cocaine. On the contrary, JWH-018 and AKB48 were ineffective on [^3^H]-DA uptake at the tested concentrations (100 nM and 1 µM, Figure 6).

## Discussion

The present multidisciplinary study, for the first time, directly compared the effects of JWH-018 and AKB48, with those of cocaine and amphetamine, to provide further insights on the mechanism of action possibly underlying the psychomotor stimulant effects of SCBs.

The behavioral studies, first, showed that JWH-018 (0.3 mg/kg) e AKB48 (1 mg/kg) facilitated spontaneous locomotion in mice through CB_1_ receptor- and DA-dependent mechanisms. In fact, the motor facilitation induced by the two SCBs was prevented by the CB_1_ receptor antagonist AM-251 as well as by the simultaneous blockade of DA D_1_ and D_2_ receptors. The SCBs-induced motor facilitation probably occurs in a narrow range of doses since SCBs mainly inhibited both spontaneous and stimulated motor activity in CD-1 mice (6, 7, 10, 41, 42). Motor impairment is one of the main behavioral effects observed after systemic administration of cannabinoid receptor agonists (43, 44), and it has been associated with the stimulation of cerebellum and basal ganglia CB_1_ receptors (43, 45, 46). However, preclinical studies reported that cannabinoid receptor agonists time- and dose-dependently modulated rodent spontaneous locomotion in a biphasic fashion, with a facilitation and an inhibition at low and high doses, respectively. This biphasic effect has been displayed by the endocannabinoid anandamide (47), Δ^9^-THC (41, 48) along with the synthetic compounds WIN 55,212-2 (44), JWH-018-R (17), 5 F-ADBINACA, AB-FUBINACA, and STS-135 (42), suggesting that it is typical of the cannabinoid system and not of a single molecule class (43).

Although the acute administration of either JWH-018 (0.3 mg/kg) or AKB48 (1 mg/kg) induced a prompt facilitation of mouse spontaneous locomotion, the profile of action of the two compounds is different. In particular, while the effect of JWH-018 is long-lasting, AKB48 only induces a transitory (15 min) increase, after which the inhibitory effect of the compound prevails, as evidenced by the significant increase in the animal’s immobility time (Figure 1B). These diverse profiles are probably due to the different doses of JWH-018 (0.3 mg/kg) and AKB48 (1 mg/kg) used, and to their pharmacokinetic, rather than pharmacodynamics, properties (*see also below*). It seems likely that the steric hindrance of the adamantly group of AKB48 delays the passage through the blood–brain barrier or limits a quick bond to CB_1_ receptors. Furthermore, although JWH-018 [Ki = 5.82 nM; (6)] and AKB48 [Ki = 5.34 nM; (7)] show similar nanomolar affinity for CD-1 mouse CB_1_ receptor, their *in vivo* behavioral responses are quantitatively different, being JWH-018 more effective (7).

Normally, rodents tend to move in the perimeter of an arena (i.e., thigmotaxis), thus, spending there more time than in the center of the apparatus. As from an ethological point of view, a mouse that spends more time in an open space is less concerned about being attacked by predators. In fact, the animal’s occupancy of the peripheral areas, either in corners or near the walls, has been identified as an index of “timidity” (49) or “anxiety” (50, 51). The present behavioral data also demonstrate that JWH-018 and AKB48 qualitatively increase the mouse spontaneous motor activity (total distance traveled) in a similar way to cocaine (18–22) and amphetamine (23–25). However, in respect to cocaine and amphetamine, the two SCBs displayed a different behavioral profile as assessed by evaluating the mouse arena’s exploration. In fact, unlike the two psychostimulants, SCBs increase the animal’s standing time at the center of the arena, suggesting an “anxiolytic-like” profile in the open field context (52, 53). This behavior, unusual for the mouse, suggests that the administration of SCBs may cause a reduction in the danger perception (54). This finding is in line with previous data demonstrating that CB_1_ receptor agonists, at least at low doses, induced anxiolytic effects in rodents (52, 55–57). However, it cannot be ruled out that the motor stimulation effect associated with motor sensory impairment caused by JWH-018 and AKB48 (7, 41) may lead the mouse to a loss of sensory contact with the walls of the box and to the consequent disoriented movements into the open space of the arena. In fact, spatial information collected by tactile sensations and integrated in visual control in rodents play a pivotal role of spatial orientation (58, 59). Conversely, cocaine and amphetamine increase the time spent in the peripheral arena, suggesting an “anxiogenic-like” effect, which is typical of stimulant substances promoting catecholaminergic transmission (60, 61). This anxiogenic-like behavior causes greater alertness and attention in the mouse by promoting the combat and flight behavior that is typical of non-predatory animals, such as the mouse (54).

As reported above, JWH-018- and AKB48-induced increases in motor activity were prevented by pretreatment with SCH23390 (D_1/5_ receptor antagonist) and haloperidol (D_2/3_ receptor antagonist), thus suggesting that increased DA transmission underlies the SCBs motor-stimulant properties. This is consistent with the implication of dopaminergic mechanisms in the motor-stimulant properties of amphetamine and cocaine (19, 62, 63). In view of this, along with the different behavioral profile of action of the compounds under investigation, *in vivo* and *in vitro* experiments have been performed in order to evaluate their effects on dopaminergic system. Interestingly, *in vivo* DaTSCAN imaging studies demonstrated that, similarly to amphetamine and cocaine, either JWH-018 or AKB48 administration decreased the [^123^I]-FP-CIT binding to DAT in mice striatum. In consideration of this finding, *in vitro* experiments have been performed to verify the possible direct interaction between the two SBCs and DAT. In fact, previous data proposed that both cannabinoid agonists and antagonists inhibit DAT activity *via* molecular targets other than CB_1_ receptors (64). The present *in vitro* competition binding experiments clearly indicated that, unlike cocaine and amphetamine, JWH-018 and AKB48 did not bind to DAT expressed in mouse striatal nerve terminals, while they showed only a low affinity (micrometer range) for human DAT in CHO transfect cell membranes. The affinity values of cocaine and amphetamine for human DAT, observed in this present study, are in line with literature data (65, 66). Despite various paper reported the affinity values of GBR 12783, cocaine, and amphetamine in rat striatum, this is the first study, to our knowledge, reporting [^3^H]-WIN 35,428 competition binding experiments of these compounds in mouse striatal synaptosomes, where they show affinity values similar to those found on human DAT. In line with the binding results, this study also demonstrates that, in contrast to cocaine and amphetamine, neither JWH-018 nor AKB48, at the concentration tested, significantly affected [^3^H]-DA uptake from murine striatal synaptosomes. This is in apparent contrast with some literature data showing that cannabinoids significantly reduces DA uptake in striatal nerve terminals or slices (64, 67, 68). However, in line with the present results, a previous study (69) failed to observe alterations of DA uptake following treatment of mouse striatal synaptosomes with some SCBs. Although other possibility cannot be definitely ruled out, it seems likely that these discrepancies could be due to the different experimental conditions used in the reported studies (i.e., different cannabinoid receptor agonists, different drug concentrations, different DA concentration, and time of incubation).

Taking into account the above *in vitro* results, the possibility that the observed JWH-018- or AKB48-induced reduction of [^123^I]-FP-CIT signal in the mice striatum is due to a direct interaction between the SCBs and DAT seems unlikely. A logical alternative explanation is that JWH-018 or AKB48 systemic administration induces an increase in the levels of endogenous DA which, in turn, competes with [^123^I]-FP-CIT for DAT. This hypothesis is supported by the present *in vivo* microdialysis results, showing that the systemic administration of a low dose of JWH-018 (0.3 mg/kg) or AKB48 (0.3 mg/kg) stimulated extracellular DA levels in the NAc shell of freely moving mice. In particular, either JWH-018 or AKB48 caused a maximal increase to ~150% of baseline DA concentrations. However, in line with the drug behavioral profile, the effect of JWH-018 was long-lasting, while the effect of AKB48 was transient. As expected, either cocaine or amphetamine also increased DA extracellular levels and their effects were significantly higher than those of the two SCBs. It is well established that the mechanism of action of these classes of drugs is different. Indeed, classical psychostimulants, as cocaine and amphetamine, increase DA neurotransmission by inhibiting the DAT activity in DA nigrostriatal and mesolimbic neuronal terminals; in particular the psychostimulant-induced increase in DA neurotransmission is mainly due to DA reuptake inhibition, an enhancement of DA release or to a combination of the two mechanisms (70–78). On the contrary, SCBs increase NAc shell DA release mainly through indirect CB_1_ receptor-mediated mechanisms. In fact, while CB_1_ receptors are not expressed on midbrain DA neurons (79), CB_1_ receptor activation closely modulates DA neuronal activity, through modulation of local circuitry in the midbrain (80). In mesolimbic DA pathway, CB_1_ receptors are located in axon terminals forming either inhibitory or excitatory-type synapses with dopaminergic as well as non-dopaminergic, putative GABAergic, neurons in the VTA, and systemic administration of CB_1_ receptor agonists enhances the bursting activity of VTA DA neurons, many of which project to the NAc shell (81). It has been reported that, by reducing the activity of GABAergic terminals, cannabinoids can facilitate dopaminergic activity through suppression of inhibitory input onto GABA_A_ or GABA_B_ receptors on DA neurons (80). In line with this, *ex vivo* whole cell patch clamp recordings from rat VTA DA neurons showed that JWH-018 decreases GABA_A_-mediated post-synaptic currents, suggesting that the stimulation of DA release observed *in vivo* might result from a disinhibition of DA neurons (26, 82, 83). The different mechanisms underlying the SBCs- or psychostimulants-induced DA release are confirmed by the present *in vitro* studies on striatum, including NAc, nerve ending. In fact, accordingly to their direct or indirect inhibitory modulation of DAT activity and DA-releasing effects, either amphetamine or cocaine significantly increased [^3^H]-DA efflux from mouse striatal synaptosomes. In this context, it is worth noting that under the present experimental conditions (i.e., 0.3 ml/min flow rate) DA levels in the perfusate have been reported to represent the net consequence of [^3^H]-DA release and reuptake (84). Differently, JWH-018 and AKB48 did not induce any effects on spontaneous [^3^H]-DA efflux from murine striatal synaptosomes. These findings are in line with previous data showing that the CB_1_/CB_2_ cannabinoid receptor agonists WIN 55,212-2 and CP 55,940 had no effects on basal and electrically evoked DA release in the corpus striatum and the NAc slices (85). The lack of a presynaptic effect on terminals of nigrostriatal and mesolimbic dopaminergic neurons is also in accord with the absence of CB_1_ receptor on dopaminergic terminals (*see above*). Taken together, these findings indicate that, at least at the concentration tested, the two SCBs did not affect the DAT activity, leading to hypothesize that their inhibitory effects on the [^123^I]-FP-CIT binding to DAT in the mice striatum could be a consequence of an increase in endogenous DA levels.

## Conclusion

The present data demonstrate, for the first time, that JWH-018 and AKB48 induce psychostimulant effects in mice possibly related to the facilitation of NAc DA release induced by the two compounds. Although the motor activation induced by the tested SCBs or the two classical psychostimulants involve dopaminergic mechanisms, it seems likely that the two classes of compound recruit different neurochemical pathways in mouse nigrostriatal and mesolimbic regions. These data, according to clinical reports, outline the potential psychostimulant action of SCBs highlighting their possible danger to human health (16, 86–89).

## Ethics Statement

All applicable international, national, and/or institutional guidelines for the care and use of animals were followed. All procedures performed in the studies involving animals were in accordance with the ethical standards of the institution or practice at which the studies were conducted. In particular, the experimental protocols performed in this study were in accordance with the new European Communities Council Directive of September 2010 (2010/63/EU) a revision of the Directive 86/609/EEC and were approved by the Italian Ministry of Health and by the Ethical Committee of the University of Ferrara and of the University of Cagliari (microdialysis studies).

## Author Contributions

Substantial contributions to the conception (AO, MM, LF, and MDL) or design of the work (AO, MM, LF, MDL, and KV); or the acquisition (AO, LU, SBilel, IC, GD, MP, GP, MDL, FV, and SBeggiato), analysis (AB, CR, PB, FD-G, and GS), or interpretation of data for the work (AO, MM, LF, and MDL); drafting the work or revising it critically for important intellectual content (AO, LU, SBilel, IC, GD, MP, GP, MDL, AB, FV, CR, SBeggiato, LF, KV, PB, GSF, F-DG, and MM); final approval of the version to be published (AO, LU, SBilel, IC, GD, MP, GP, MDL, AB, FV, CR, SBeggiato, LF, KV, PB, GF, FD-G, and MM); and agreement to be accountable for all aspects of the work in ensuring that questions related to the accuracy or integrity of any part of the work are appropriately investigated and resolved (AO, LU, SBilel, IC, GD, MP, GP, MDL, AB, FV, CR, SBeggiato, LF, KV, PB, GS, FD-G, and MM).

## Conflict of Interest Statement

The authors declare that the research was conducted in the absence of any commercial or financial relationships that could be construed as a potential conflict of interest.

## Abbreviations

AKB48, *N*-(1-adamantyl)-1-pentyl-1H-indazole-3-carboxamide; DA, dopamine; DAT, dopamine transporter; JWH-018, naphthalen-1-yl-(1-pentylindol-3-yl)methanone; HAL, 4-[4-(4-chlorophenyl)-4-hydroxypiperidin-1-yl]-1-(4-fluorophenyl)butan-1-one; haloperidol; GBR 12783, 1-(2-benzhydryloxyethyl)-4-[(E)-3-phenylprop-2-enyl]piperazine;dihydrochloride; NAc shell, nucleus accumbens shell; [^123^I]FP-CIT, (^123^I-2β-carbomethoxy-3β-(4-iodophenyl)-*N*-(3-fluoropropyl)nortropane); SCH23390, 8-chloro-3-methyl-5-phenyl-1,2,4,5-tetrahydro-3-benzazepin-7-ol.

## References

[1] EMCDDA. EU Drug Markets Report: In-Depth Analysis. Luxembourg: EMCDDA–Europol Joint Publications, Publications Office of the European Union (2016). Available from: http://www.emcdda.europa.eu/publications/joint-publications/eu-drug-markets-2016-in-depth-analysis

[2] EMCDDA. European Monitoring Centre for Drugs and Drug Addiction, European Drug Report 2016: Trends and Developments. Luxembourg: Publications Office of the European Union (2016). Available from: http://www.emcdda.europa.eu/system/files/publications/2637/TDAT16001ENN.pdf

[3] White CM. The pharmacologic and clinical effects of illicit synthetic cannabinoids. J Clin Pharmacol (2017) 57(3):297–304. 10.1002/jcph.827 27610597

[4] European Monitoring Centre for Drugs and Drug Addiction. Thematic Paper—Understanding the ‘Spice’ Phenomenon. Lisbon, Portugal: European Monitoring Centre for Drugs and Drug Addiction (2009).

[5] NFLIS. Annual Report. (2013). Available from: http://www.deadiversion.usdoj.gov/nflis/NFLIS2013AR.pdf

[6] Vigolo A Ossato A Trapella C Vincenzi F Rimondo C Seri C Novel halogenated derivates of JWH-018: behavioral and binding studies in mice. Neuropharmacology (2015) 95:68–82. 10.1016/j.neuropharm.2015.02.008 25769232

[7] Canazza I Ossato A Trapella C Fantinati A De Luca MA Margiani G Effect of the novel synthetic cannabinoids AKB48 and 5F-AKB48 on “tetrad”, sensorimotor, neurological and neurochemical responses in mice. In vitro and in vivo pharmacological studies. Psychopharmacology (2016) 233(21–22):3685–709. 10.1007/s00213-016-4402-y 27527584

[8] Koob GF Volkow ND Neurocircuitry of addiction. Neuropsychopharmacology (2010) 35(1):217–38. 10.1038/npp.2009.110 19710631 PMC2805560

[9] De Luca MA Bimpisidis Z Melis M Marti M Caboni P Valentini V Stimulation OF IN VIVO dopamine transmission and intravenous self-administration in rats and mice by JWH-018, a spice cannabinoid. Neuropharmacology (2015) 99:705–14. 10.1016/j.neuropharm.2015.08.041 26327678

[10] Ossato A Canazza I Trapella C Vincenzi F De Luca MA Rimondo C Effect of JWH-250, JWH-073 and their interaction on “tetrad”, sensorimotor, neurological and neurochemical responses in mice. Prog Neuropsychopharmacol Biol Psychiatry (2016) 67:31–50. 10.1016/j.pnpbp.2016.01.007 26780169

[11] Di Chiara G Bassareo V Fenu S De Luca MA Spina L Cadoni C Dopamine and drug addiction: the nucleus accumbens shell connection. Neuropharmacology (2004) 47(Suppl 1):227–41. 10.1016/j.neuropharm.2004.06.032 15464140

[12] Miliano C Serpelloni G Rimondo C Mereu M Marti M De Luca MA. Neuropharmacology of new psychoactive substances (NPS): focus on the rewarding and reinforcing properties of cannabimimetics and amphetamine-like stimulants. Front Neurosci (2016) 10:153. 10.3389/fnins.2016.00153 27147945 PMC4835722

[13] Brewer TL Collins M. A review of clinical manifestations in adolescent and young adults after use of synthetic cannabinoids. J Spec Pediatr Nurs (2014) 19(2):119–26. 10.1111/jspn.12057 24320158

[14] Tournebize J Gibaja V Kahn JP Acute effects of synthetic cannabinoids: update 2015. Subst Abuse (2016):1–23. 10.1080/08897077.2016.1219438 27715709

[15] Cooper ZD. Adverse effects of synthetic cannabinoids: management of acute toxicity and withdrawal. Curr Psychiatry Rep (2016) 18(5):52. 10.1007/s11920-016-0694-1 27074934 PMC4923337

[16] Winstock AR Barratt MJ. Synthetic cannabis: a comparison of patterns of use and effect profile with natural cannabis in a large global sample. Drug Alcohol Depend (2013) 131(1–2):106–11. 10.1016/j.drugalcdep.2012.12.011 23291209

[17] Barbieri M Ossato A Canazza I Trapella C Borelli AC Beggiato S Synthetic cannabinoid JWH-018 and its halogenated derivatives JWH-018-Cl and JWH-018-Br impair novel object recognition in mice: behavioral, electrophysiological and neurochemical evidence. Neuropharmacology (2016) 109:254–69. 10.1016/j.neuropharm.2016.06.027 27346209

[18] Zubrycki EM Giordano M Sanberg PR. The effects of cocaine on multivariate locomotor behavior and defecation. Behav Brain Res (1990) 36(1–2):155–9. 10.1016/0166-4328(90)90169-F 2302315

[19] Broderick PA Rahni DN Zhou Y. Acute and subacute effects of risperidone and cocaine on accumbens dopamine and serotonin release using in vivo microvoltammetry on line with open-field behavior. Prog Neuropsychopharmacol Biol Psychiatry (2003) 27(6):1037–54. 10.1016/s0278-5846(03)00176-3 14499322

[20] Jiang Q Wang CM Fibuch EE Wang JQ Chu XP. Differential regulation of locomotor activity to acute and chronic cocaine administration by acid-sensing ion channel 1a and 2 in adult mice. Neuroscience (2013) 246:170–8. 10.1016/j.neuroscience.2013.04.059 23644053 PMC3855427

[21] Simchon-Tenenbaum Y Weizman A Rehavi M. Alterations in brain neurotrophic and glial factors following early age chronic methylphenidate and cocaine administration. Behav Brain Res (2015) 282:125–32. 10.1016/j.bbr.2014.12.058 25576963

[22] Zombeck JA Swearingen SP Rhodes JS. Acute locomotor responses to cocaine in adolescents vs. adults from four divergent inbred mouse strains. Genes Brain Behav (2010) 9(8):892–8. 10.1111/j.1601-183X.2010.00630.x 20662938 PMC2975896

[23] Sanberg PR Henault MA Hagenmeyer-Houser SH Russell KH. The topography of amphetamine and scopolamine-induced hyperactivity: toward an activity print. Behav Neurosci (1987) 101(1):131–3. 10.1037/0735-7044.101.1.131 3828051

[24] Laviola G Dell’Omo G Chiarotti F Bignami G. d-Amphetamine conditioned place preference in developing mice: relations with changes in activity and stereotypies. Behav Neurosci (1994) 108(3):514–24. 10.1037/0735-7044.108.3.514 7917045

[25] Proietti Onori M Ceci C Laviola G Macri S. A behavioural test battery to investigate tic-like symptoms, stereotypies, attentional capabilities, and spontaneous locomotion in different mouse strains. Behav Brain Res (2014) 267:95–105. 10.1016/j.bbr.2014.03.023 24675156

[26] De Luca MA Castelli MP Loi B Porcu A Martorelli M Miliano C Native CB1 receptor affinity, intrinsic activity and accumbens shell dopamine stimulant properties of third generation SPICE/K2 cannabinoids: BB-22, 5F-PB-22, 5F-AKB-48 and STS-135. Neuropharmacology (2016) 105:630–8. 10.1016/j.neuropharm.2015.11.017 26686391

[27] Cheng MH Block E Hu F Cobanoglu MC Sorkin A Bahar I Insights into the modulation of dopamine transporter function by amphetamine, orphenadrine, and cocaine binding. Front Neurol (2015) 6:134 10.3389/fneur.2015.00134 26106364 PMC4460958

[28] Ferraro L Beggiato S Marcellino D Frankowska M Filip M Agnati LF Nanomolar concentrations of cocaine enhance D2-like agonist-induced inhibition of the K+-evoked [3H]-dopamine efflux from rat striatal synaptosomes: a novel action of cocaine. J Neural Transm (Vienna) (2010) 117(5):593–7. 10.1007/s00702-010-0389-4 20354886

[29] Binda F Dipace C Bowton E Robertson SD Lute BJ Fog JU Syntaxin 1A interaction with the dopamine transporter promotes amphetamine-induced dopamine efflux. Mol Pharmacol (2008) 74(4):1101–8. 10.1124/mol.108.048447 18617632 PMC2728020

[30] Del Guerra A Bartoli A Belcari N Herbert D Motta A Vaiano A Performance evaluation of the fully engineered YAP-(S)PET scanner for small animal imaging. IEEE Trans Nucl Sci (2006) 53:1078–83. 10.1109/TNS.2006.871900

[31] Boschi A Pasquali M Uccelli L Duatti A Novel Tc-99m radiotracers for brain imaging. Braz Arch Biol Technol (2007) 50:37–44. 10.1590/S1516-89132007000600005

[32] Esposito E Boschi A Ravani L Cortesi R Drechsler M Mariani P Biodistribution of nanostructured lipid carriers: a tomographic study. Eur J Pharm Biopharm (2015) 89:145–56. 10.1016/j.ejpb.2014.12.006 25497177

[33] Smilkov K Janevik E Guerrini R Pasquali M Boschi A Uccelli L Preparation and first biological evaluation of novel Re-188/Tc-99m peptide conjugates with substance-P. Appl Radiat Isot (2014) 92:25–31. 10.1016/j.apradiso.2014.06.003 24973465

[34] NEMA. Performance Measurements of Small Animal Positron Emission Tomographs. Standard Publication NU 4-2008. Rosslyn, VA: National Electrical Manufacturers Association (2008).

[35] Cittanti C Uccelli L Pasquali M Boschi A Flammia C Bagatin E Whole-body biodistribution and radiation dosimetry of the new cardiac tracer 99mTc-N-DBODC. J Nucl Med (2008) 49(8):1299–304. 10.2967/jnumed.108.053132 18632816

[36] Di Domenico G Cesca N Zavattini G Auricchi N Gambaccini M CT with a CMOS flat panel detector integrated on the YAP(S)PET scanner for in vivo small animal imaging. Nucl Instrum Methods Phys Res (2007) 571:110–3. 10.1016/j.nima.2006.10.042

[37] Loening AM Gambhir SS. AMIDE: a free software tool for multimodality medical image analysis. Mol Imaging (2003) 2(3):131–7. 10.1162/153535003322556877 14649056

[38] MacKenzie-Graham A Boline J Toga AW. Brain atlases and neuroanatomic imaging. Methods Mol Biol (2007) 401:183–94. 10.1007/978-1-59745-520-6_11 18368367

[39] Ma Y Hof PR Grant SC Blackband SJ Bennett R Slatest L A three-dimensional digital atlas database of the adult C57BL/6J mouse brain by magnetic resonance microscopy. Neuroscience (2005) 135(4):1203–15. 10.1016/j.neuroscience.2005.07.014 16165303

[40] Paxinos G Franklin KBJ The Mouse Brain in Stereotaxic Coordinates. San Diego: Academic Press (2001).

[41] Ossato A Vigolo A Trapella C Seri C Rimondo C Serpelloni G JWH-018 impairs sensorimotor functions in mice. Neuroscience (2015) 300:174–88. 10.1016/j.neuroscience.2015.05.021 25987201

[42] Canazza I Ossato A Vincenzi F Gregori A Di Rosa F Nigro F Pharmaco-toxicological effects of the novel third-generation fluorinate synthetic cannabinoids, 5F-ADBINACA, AB-FUBINACA, and STS-135 in mice. In vitro and in vivo studies. Hum Psychopharmacol (2017) 32:e2601. 10.1002/hup.2601 28597570

[43] Rodriguez de Fonseca F Del Arco I Martin-Calderon JL Gorriti MA Navarro M Role of the endogenous cannabinoid system in the regulation of motor activity. Neurobiol Dis (1998) 5(6 Pt B):483–501. 9974180 10.1006/nbdi.1998.0217

[44] Drews E Schneider M Koch M. Effects of the cannabinoid receptor agonist WIN 55,212-2 on operant behavior and locomotor activity in rats. Pharmacol Biochem Behav (2005) 80(1):145–50. 10.1016/j.pbb.2004.10.023 15652390

[45] Breivogel CS Childers SR. The functional neuroanatomy of brain cannabinoid receptors. Neurobiol Dis (1998) 5(6 Pt B):417–31. 10.1006/nbdi.1998.0229 9974175

[46] Sanudo-Pena MC Tsou K Walker JM. Motor actions of cannabinoids in the basal ganglia output nuclei. Life Sci (1999) 65(6–7):703–13. 10.1016/S0024-3205(99)00293-3 10462071

[47] Sulcova E Mechoulam R Fride E. Biphasic effects of anandamide. Pharmacol Biochem Behav (1998) 59(2):347–52. 10.1016/S0091-3057(97)00422-X 9476980

[48] Katsidoni V Kastellakis A Panagis G. Biphasic effects of Δ9-tetrahydrocannabinol on brain stimulation reward and motor activity. Int J Neuropsychopharmacol (2013) 16:2273–84. 10.1017/S1461145713000709 23830148

[49] Walsh RN Cummins RA The open-field test: a critical review. Psychol Bull (1976) 83(3):482–504. 10.1037/0033-2909.83.3.482 17582919

[50] Simon P Dupuis R Costentin J. Thigmotaxis as an index of anxiety in mice. Influence of dopaminergic transmissions. Behav Brain Res (1994) 61(1):59–64. 10.1016/0166-4328(94)90008-6 7913324

[51] Treit D Fundytus M. Thigmotaxis as a test for anxiolytic activity in rats. Pharmacol Biochem Behav (1988) 31(4):959–62. 10.1016/0091-3057(88)90413-3 3252289

[52] Haller J Varga B Ledent C Freund TF. CB1 cannabinoid receptors mediate anxiolytic effects: convergent genetic and pharmacological evidence with CB1-specific agents. Behav Pharmacol (2004) 15(4):299–304. 10.1097/01.fbp.0000135704.56422.40 15252281

[53] Macri S Lanuzza L Merola G Ceci C Gentili S Valli A Behavioral responses to acute and sub-chronic administration of the synthetic cannabinoid JWH-018 in adult mice prenatally exposed to corticosterone. Neurotox Res (2013) 24(1):15–28. 10.1007/s12640-012-9371-2 23296549

[54] Ennaceur A Chazot PL. Preclinical animal anxiety research – flaws and prejudices. Pharmacol Res Perspect (2016) 4(2):e00223. 10.1002/prp2.223 27069634 PMC4804324

[55] Rey AA Purrio M Viveros MP Lutz B. Biphasic effects of cannabinoids in anxiety responses: CB1 and GABA(B) receptors in the balance of GABAergic and glutamatergic neurotransmission. Neuropsychopharmacology (2012) 37(12):2624–34. 10.1038/npp.2012.123 22850737 PMC3473327

[56] Kinden R Zhang X Cannabinoids & stress: impact of HU-210 on behavioral tests of anxiety in acutely stressed mice. Behav Brain Res (2015) 284:225–30. 10.1016/j.bbr.2015.02.025 25707713

[57] Flores A Julia-Hernandez M Maldonado R Berrendero F Involvement of the orexin/hypocretin system in the pharmacological effects induced by delta(9)-tetrahydrocannabinol. Br J Pharmacol (2016) 173(8):1381–92. 10.1111/bph.13440 26799708 PMC4940815

[58] Brecht M Preilowski B Merzenich MM. Functional architecture of the mystacial vibrissae. Behav Brain Res (1997) 84(1–2):81–97. 10.1016/S0166-4328(97)83328-1 9079775

[59] Mitchinson B Prescott TJ. Whisker movements reveal spatial attention: a unified computational model of active sensing control in the rat. PLoS Comput Biol (2013) 9(9):e1003236. 10.1371/journal.pcbi.1003236 24086120 PMC3784505

[60] Yang XM Gorman AL Dunn AJ Goeders NE. Anxiogenic effects of acute and chronic cocaine administration: neurochemical and behavioral studies. Pharmacol Biochem Behav (1992) 41(3):643–50. 10.1016/0091-3057(92)90386-T 1584846

[61] Lin HQ Burden PM Christie MJ Johnston GA. The anxiogenic-like and anxiolytic-like effects of MDMA on mice in the elevated plus-maze: a comparison with amphetamine. Pharmacol Biochem Behav (1999) 62(3):403–8. 10.1016/S0091-3057(98)00191-9 10080230

[62] Cabib S Castellano C Cestari V Filibeck U Puglisi-Allegra S. D1 and D2 receptor antagonists differently affect cocaine-induced locomotor hyperactivity in the mouse. Psychopharmacology (1991) 105(3):335–9. 10.1007/BF02244427 1839178

[63] Gold LH Geyer MA Koob GF. Neurochemical mechanisms involved in behavioral effects of amphetamines and related designer drugs. NIDA Res Monogr (1989) 94:101–26. 2514360

[64] Price DA Owens WA Gould GG Frazer A Roberts JL Daws LC CB1-independent inhibition of dopamine transporter activity by cannabinoids in mouse dorsal striatum. J Neurochem (2007) 101(2):389–96. 10.1111/j.1471-4159.2006.04383.x 17250681

[65] Pristupa ZB Wilson JM Hoffman BJ Kish SJ Niznik HB Pharmacological heterogeneity of the cloned and native human dopamine transporter: disassociation of [3H]WIN 35,428 and [3H]GBR 12,935 binding. Mol Pharmacol (1994) 45(1):125–35. 8302271

[66] Iversen L Gibbons S Treble R Setola V Huang XP Roth BL. Neurochemical profiles of some novel psychoactive substances. Eur J Pharmacol (2013) 700(1–3):147–51. 10.1016/j.ejphar.2012.12.006 23261499 PMC3582025

[67] Howes J Osgood P The effect of delta9-tetrahydrocannabinol on the uptake and release of 14C-dopamine from crude striatal synaptosoma; preparations. Neuropharmacology (1974) 13(12):1109–14. 10.1016/0028-3908(74)90060-4 4457762

[68] Banerjee SP Snyder SH Mechoulam R. Cannabinoids: influence on neurotransmitter uptake in rat brain synaptosomes. J Pharmacol Exp Ther (1975) 194(1):74–81. 168349

[69] Kofalvi A Rodrigues RJ Ledent C Mackie K Vizi ES Cunha RA Involvement of cannabinoid receptors in the regulation of neurotransmitter release in the rodent striatum: a combined immunochemical and pharmacological analysis. J Neurosci (2005) 25(11):2874–84. 10.1523/jneurosci.4232-04.2005 15772347 PMC6725145

[70] De Wit H Wise RA Blockade of cocaine reinforcement in rats with the dopamine receptor blocker pimozide, but not with the noradrenergic blockers phentolamine or phenoxybenzamine. Can J Psychol (1977) 31(4):195–203. 10.1037/h0081662 608135

[71] Church WH Justice JB Jr Byrd LD. Extracellular dopamine in rat striatum following uptake inhibition by cocaine, nomifensine and benztropine. Eur J Pharmacol (1987) 139(3):345–8. 10.1016/0014-2999(87)90592-9 3666010

[72] Ritz MC Cone EJ Kuhar MJ. Cocaine inhibition of ligand binding at dopamine, norepinephrine and serotonin transporters: a structure-activity study. Life Sci (1990) 46(9):635–45. 10.1016/0024-3205(90)90132-B 2308472

[73] Bradberry CW Roth RH. Cocaine increases extracellular dopamine in rat nucleus accumbens and ventral tegmental area as shown by in vivo microdialysis. Neurosci Lett (1989) 103(1):97–102. 10.1016/0304-3940(89)90492-8 2779859

[74] Hurd YL Ungerstedt U. Ca2+ dependence of the amphetamine, nomifensine, and Lu 19-005 effect on in vivo dopamine transmission. Eur J Pharmacol (1989) 166(2):261–9. 10.1016/0014-2999(89)90067-8 2477260

[75] Kalivas PW Duffy P Effect of acute and daily cocaine treatment on extracellular dopamine in the nucleus accumbens. Synapse (1990) 5(1):48–58. 10.1002/syn.890050104 2300906

[76] Broderick PA. Cocaine: on-line analysis of an accumbens amine neural basis for psychomotor behavior. Pharmacol Biochem Behav (1991) 40(4):959–68. 10.1016/0091-3057(91)90112-F 1816582 PMC7133205

[77] Broderick PA. In vivo electrochemical studies of gradient effects of (SC) cocaine on dopamine and serotonin release in dorsal striatum of conscious rats. Pharmacol Biochem Behav (1993) 46(4):973–84. 10.1016/0091-3057(93)90231-H 8309978

[78] Broderick PA Kornak EP Jr Eng F Wechsler R. Real time detection of acute (IP) cocaine-enhanced dopamine and serotonin release in ventrolateral nucleus accumbens of the behaving Norway rat. Pharmacol Biochem Behav (1993) 46(3):715–22. 10.1016/0091-3057(93)90567-D 8278450 PMC7133218

[79] Julian MD Martin AB Cuellar B Rodriguez De Fonseca F Navarro M Moratalla R Neuroanatomical relationship between type 1 cannabinoid receptors and dopaminergic systems in the rat basal ganglia. Neuroscience (2003) 119(1):309–18. 10.1016/S0306-4522(03)00070-8 12763090

[80] Covey DP Mateo Y Sulzer D Cheer JF Lovinger DM Endocannabinoid modulation of dopamine neurotransmission. Neuropharmacology (2017). 10.1016/j.neuropharm.2017.04.033 PMC5608040 28450060

[81] Fitzgerald ML Shobin E Pickel VM. Cannabinoid modulation of the dopaminergic circuitry: implications for limbic and striatal output. Prog Neuropsychopharmacol Biol Psychiatry (2012) 38(1):21–9. 10.1016/j.pnpbp.2011.12.004 22265889 PMC3389172

[82] Melis M Sagheddu C De Felice M Casti A Madeddu C Spiga S Enhanced endocannabinoid-mediated modulation of rostromedial tegmental nucleus drive onto dopamine neurons in Sardinian alcohol-preferring rats. J Neurosci (2014) 34(38):12716–24. 10.1523/jneurosci.1844-14.2014 25232109 PMC4166158

[83] Melis M Frau R Kalivas PW Spencer S Chioma V Zamberletti E New vistas on cannabis use disorder. Neuropharmacology (2017). 10.1016/j.neuropharm.2017.03.033 28373077 PMC5865400

[84] Bowyer JF Masserano JM Weiner N Inhibitory effects of amphetamine on potassium-stimulated release of [3H]dopamine from striatal slices and synaptosomes. J Pharmacol Exp Ther (1987) 240(1):177–86. 3100768

[85] Szabo B Muller T Koch H. Effects of cannabinoids on dopamine release in the corpus striatum and the nucleus accumbens in vitro. J Neurochem (1999) 73(3):1084–9. 10.1046/j.1471-4159.1999.0731084.x 10461898

[86] Hermanns-Clausen M Kneisel S Szabo B Auwarter V. Acute toxicity due to the confirmed consumption of synthetic cannabinoids: clinical and laboratory findings. Addiction (2013) 108(3):534–44. 10.1111/j.1360-0443.2012.04078.x 22971158

[87] Gurney SM Scott KS Kacinko SL Presley BC Logan BK. Pharmacology, toxicology, and adverse effects of synthetic cannabinoid drugs. Forensic Sci Rev (2014) 26:53–78. 26226970

[88] Fattore L Synthetic cannabinoids-further evidence supporting the relationship between cannabinoids and psychosis. Biol Psychiatry (2016) 79(7):539–48. 10.1016/j.biopsych.2016.02.001 26970364

[89] Muller HH Kornhuber J Sperling W. The behavioral profile of spice and synthetic cannabinoids in humans. Brain Res Bull (2016) 126(Pt 1):3–7. 10.1016/j.brainresbull.2015.10.013 26548494

